# External Pressure in Polymer-Based Lithium Metal Batteries: An Often-Neglected Criterion When Evaluating Cycling Performance?

**DOI:** 10.1021/acsami.4c02095

**Published:** 2024-04-22

**Authors:** Philipp Roering, Gerrit Michael Overhoff, Kun Ling Liu, Martin Winter, Gunther Brunklaus

**Affiliations:** † Helmholtz-Institute Münster, IEK-12, Forschungszentrum Jülich GmbH, Corrensstraße 46, 48149 Münster, Germany; ‡ MEET Battery Research Center/Institute of Physical Chemistry, University of Münster, Corrensstraße 46, 48149 Münster, Germany

**Keywords:** solid-state battery, lithium metal battery, solid polymer electrolyte, pressure, mechanical stability, poly(ethylene oxide)

## Abstract

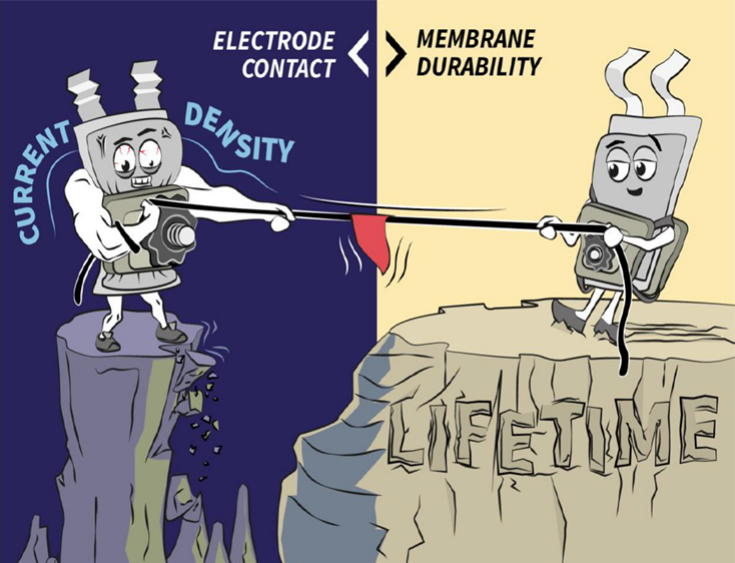

Solid-state batteries based on lithium metal anodes, solid electrolytes, and composite cathodes constitute a promising battery concept for achieving high energy density. Charge carrier transport within the cells is governed by solid–solid contacts, emphasizing the importance of well-designed interfaces. A key parameter for enhancing the interfacial contacts among electrode active materials and electrolytes comprises externally applied pressure onto the cell stack, particularly in the case of ceramic electrolytes. Reports exploring the impact of external pressure on polymer-based cells are, however, scarce due to overall better wetting behavior. In this work, the consequences of externally applied pressure in view of key performance indicators, including cell longevity, rate capability, and limiting current density in single-layer pouch-type NMC622||Li cells, are evaluated employing cross-linked poly(ethylene oxide), xPEO, and cross-linked cyclodextrin grafted poly(caprolactone), xGCD-PCL. Notably, externally applied pressure substantially changes the cell's electrochemical cycling performance, strongly depending on the mechanical properties of the considered polymers. Higher external pressure potentially enhances electrode–electrolyte interfaces, thereby boosting the rate capability of pouch-type cells, despite the fact that the cell longevity may be reduced upon plastic deformation of the polymer electrolytes when passing beyond intrinsic thresholds of compressive stress. For the softer xGCD-PCL membrane, cycling of cells is only feasible in the absence of external pressure, whereas in the case of xPEO, a trade-off between enhanced rate capability and minimal membrane deformation is achieved at cell pressures of ≤0.43 MPa, which is considerably lower and more practical compared to cells employing ceramic electrolytes with ≥5 MPa external pressure.

## 1 Introduction

Electrification of the mobility sector to reduce CO_2_ emissions results in a continuously growing demand for high-performance batteries and suitable cell concepts to fulfill the imposed requirements of energy densities. Despite several technical challenges, thin lithium (Li) metal anodes have (re)emerged as a promising constituent, attributed to the high theoretical energy density of up to 3860 mAh g^–1^.^1,2^ In practice, inhomogeneous Li metal deposition and associated losses of Li inventory currently impede its widespread application.^3,4^ To overcome these obstacles, research efforts to prevent inhomogeneous deposition of Li, including exploitation of solid electrolytes, increased over the last few years. These solid electrolytes, classified as inorganic ceramics or polymers, are considered a safer approach compared to liquid electrolytes for the implementation of Li metal anodes due to higher mechanical stability and the absence of volatile, flammable components.^5,6^ According to simulations based on the linear elasticity theory of Monroe and Newman, a shear modulus exceeding twice that of pristine Li metal (>2 × 2.8 GPa) should theoretically be sufficient for an electrolyte or separator to withstand dendritic lithium protrusion.^7^ The prevalent ceramic materials, oxides and sulfides, exhibit ionic conductivities of 10^–5^ up to 10^–2^ S cm^–1^,^8,9^ often affording single-ion conducting behavior and fulfilling the relevant criteria proposed by Newman and Monroe.^10^ However, several reports have stated the formation of dendritic Li species in the case of these electrolytes.^11−13^ The model is constructed based on an ideal system with homogeneous contacts between Li metal and the electrolyte, solely focusing on the elastic properties of the solid electrolyte, while in practice, the integrity of the interfaces between Li metal and the electrolyte, the presence of grain boundaries, or other inhomogeneities considerably impact dendritic Li growth. Insufficient contacts within the solid–solid interphases between the ceramic electrolyte and electrode materials may result in larger interfacial resistances coupled with inhomogeneous Li deposition. While there have been efforts to reduce interface resistances by addition of small amounts of liquid electrolytes,^14,15^ the most effective and common method to improve the interfacial contacts represents the application of a reasonably large external pressure (up to 50 MPa).^16,17^ The benefits of external pressure could be demonstrated for ceramic electrolytes, including lower interfacial resistances,^16−18^ reduced overpotentials,^19,20^ and enhanced limiting current densities,^21^ thus enabling prolonged cycle life of the cells. In academia, a press is utilized to apply external pressures to the cells (e.g., in a range of 1–10 MPa, depending on the ceramic material), but it remains a critical factor for large battery packs necessary for electric vehicle applications, thus constraining design opportunities and the energy density of the considered battery packs. Furthermore, Li metal, possessing a yield stress of 0.8 MPa, is susceptible to undergo plastic deformation and creep when subjected to excessively high external pressures.^22^ This phenomenon can result in a penetration of Li through grain boundaries of the ceramic electrolyte, thereby posing a risk of cell short-circuiting.^17^ Nevertheless, the external pressure is considered a crucial factor when evaluating cell performance, and its impact on material properties is examined rigorously.^23^

Polymer electrolytes as the second type of solid electrolytes indeed have the benefit of better electrode wettability as they are more flexible,^24^ especially at elevated temperatures (40–60 °C), thereby reducing interphase resistances.^25^ The first commercial lithium metal batteries employing polymer electrolytes were produced by Bolloré and found application in electric cars and buses, despite necessitating elevated temperatures for operational purposes due to limited ionic conductivity of the polymer electrolyte. It should be noted that even though polymers usually have limited mechanical strength (<1 MPa) and do not meet the previously mentioned criteria of Monroe and Newman, a decrease in dendritic Li growth was reported by Barai et al. based on simulations. They stated elastic-plastic deformation of the polymer electrolyte and Li metal when the electrolyte exhibited at least a shear modulus of *G*^Electrolyte^ > 10^–3^*G*^Li^ as a result of effective stress within Li metal that surpasses its yield limit.^26^ Besides mechanical strength, factors such as temperature, external pressure, ion transport capability within the electrolyte and across electrode interfaces, and the utilized current densities (fast charge) affect the actual Li metal deposition.^27^ The external pressure is typically considered less important in polymer-based Li metal batteries, and more effort is put into increasing the ionic conductivity or enhancing the electrochemical stability of the respective polymer electrolytes even though changes in Li metal deposition have already been reported for liquid-based Li metal batteries when applying external pressure to the cell stack.^28,29^ Especially for coin cells or small pouch cells, often no details regarding the actual cell pressures are mentioned in the experimental section, even though the selection of spacers, nature of Li metal anodes, or thickness of the polymer electrolyte membrane can have an impact on the cell pressure (as demonstrated in Table 1). Gupta et al. reported a decrease in interfacial resistance in Li|PEO|Li cells as stack pressures were applied, eventually stabilizing at 0.4 and 0.2 MPa for temperatures of 60 and 80 °C, respectively.^30^ Though these findings unequivocally indicate the importance of external pressure for polymer electrolytes, the impact during prolonged cycling and results for full cells with an appropriate cathode material have not yet been demonstrated.

**Table 1:** Pressure Calculations inside a CR2032 Coin Cell and Adjustment of Applied Pressure When Exchanging Cell Components

	**standard setup**^a^	**thicker (300 μm) Li metal electrode**	**thinner (25 μm) polymer membrane**	**usage of 2 1 mm spacers**
applied pressure (MPa)	0.21	0.43	0.15	0.65
change in cell pressure (%)		104	29	209

a Standard setup: 12 mm electrodes, 50 μm thick Li metal, 40 μm thick cathode, 100 μm thick polymer membrane, and exploitation of one 1 mm and one 0.5 mm spacer; details regarding the calculation are given in the Supporting Information.

In this work, the influence of external pressure onto meaningful key performance indicators such as cell longevity, rate capability, and limiting current density (LCD) was investigated. Different external pressures were applied onto single-layer pouch-type cells operated with Li metal anodes and LiNi_0.6_Mn_0.2_Co_0.2_O_2_ (NMC_622_) cathodes, and their electrochemical cycling performance, as well as the results from electrochemical impedance spectroscopy (EIS), were compared. Poly(ethylene oxide) (PEO) was utilized as a polymer electrolyte candidate as it is, to date, the most commonly explored solid electrolyte that has been utilized in commercial battery packs so far. It is found that the external pressure potentially improves the contacts between polymer electrolytes and electrodes but also yields plastic deformation of the solid polymer electrolyte (SPE), thereby locally thinning the membrane and reducing pathways for Li dendritic growth toward the positive electrode. The external pressure indeed influences the electrochemical performance, notably in cells containing polymer electrolytes. Thus, external pressure should always be considered when evaluating the electrochemical performance of the battery cell.

## 2 Experimental Section

### 2.1 Materials

Poly(ethylene oxide) (PEO, *M*_n_ = 5 000 000 g mol^–1^, Aldrich) and benzophenone (Aldrich) were dried at 40 °C under reduced pressure (10^–3^ mbar) for 5 days. Bis(trifluoromethane)-sulfonimide Li salt (LiTFSI, purity = 99.95%, Aldrich) was dried at 120 °C under reduced pressure (10^–3^ mbar) for 2 days. α-Cyclodextrin grafted poly(caprolactone) (GCD-PCL, *M*_n_ = 76 000 g mol^–1^) was synthesized according to a previous publication^31^ and dried at 40 °C under reduced pressure (10^–3^ mbar) for 5 days. PEO, GCD-PCL, benzophenone, and LiTFSI were subsequently stored inside a glovebox (MBraun Unilab, <0.1 ppm of H_2_O, <0.1 ppm of O_2_) under an inert argon atmosphere. Li metal (50 μm, Honjo Lithium) was stored in a glovebox (MBraun Unilab, <0.1 ppm of H_2_O, <0.1 ppm of O_2_) and was used without any further surface modification.

### 2.2 Solid Polymer Electrolyte (SPE) Preparation

For the preparation of the PEO-based SPE 0.605 g of PEO, 0.395 g of LiTFSI ([Li^+^]:[EO] ratio of 1:10), and 0.05 g of benzophenone (8.25 wt % with respect to the polymer weight) as cross-linker were weighed. For the preparation of the GCD-PCL-based SPE 1.0 g of GCD-PCL, 0.503 g of LiTFSI ([Li^+^]:[C=O] ratio of 1:5), and 0.01 g of benzophenone (1.00 wt % with respect to the polymer weight) as cross-linker were weighed. For both SPEs, the ingredients were mortared to obtain a homogeneous, cotton-like powder. The powder was formed into a ball and vacuum-sealed in a pouch foil, which was placed in an oven at 100 °C for PEO and at 60 °C for GCD-PCL-based electrolytes for 2 days. After removing the mixture from the pouch foil, it was hot-pressed (100 °C, 1 MPa, 5 min, followed by 100 °C, 10 MPa, 5 min for PEO; 60 °C, 1 MPa, 3 min, followed by 60 °C, 2 MPa, 5 min for GCD-PCL) to a flat membrane (100 μm). The membrane was then placed under a UV lamp (Hönle UVACUBE 100) for 5 or 20 min, respectively, to initiate the cross-linking and to form a dense network. All the work was done in a dry room (dew point = −65 °C, relative humidity = 0.022%) to avoid any contamination with moisture.

### 2.3 Cathode Preparation

For the preparation of the cathodes, 0.9 g of LiNi_0.6_Mn_0.2_Co_0.2_O_2_ (NMC_622_, BASF Toda, 90 wt %), 0.07 g of conductive carbon (SuperP, Imerys, 7 wt %) and 0.03 g of binder (poly(vinylidene fluoride) (PVdF) 1100, Kureha, 3 wt %) dissolved in 2 mL of *N*-methyl-2-pyrrolidone (NMP) were weighed in a sample container. The container was transferred to a Thinky centrifugal mixer and stirred twice for five minutes at 1700 rounds per minute. Then, the resulting homogeneous slurry was cast onto an aluminum current collector using a doctor blade technique with a gap width of 50 μm. The coating was dried in an oven at 80 °C overnight. To obtain a homogeneous thickness and surface, the cathode sheets were roll-pressed to a final thickness of ∼40 μm (20 μm aluminum current collector, 20 μm electrode coating) resulting in a mass loading of ∼1.8 mg cm^–2^. Round disks with a diameter of Ø = 12 mm or square disks with a size of 40 × 40 mm were punched out and dried at 120 °C under reduced pressure (10^–3^ mbar) prior to use.

### 2.4 Cell Assembly

#### 2.4.1 Coin Cells

For the measurement of the LCD, Li||Li symmetric cells were assembled. A coin-cell-type (CR2032) two-electrode setup was applied with two Li metal discs (Ø = 13 mm) separated by the selected SPE (thickness = ∼100 μm, Ø = 14 mm). For comparison between the different cell setups, also NMC_622_||Li cells were assembled, where an NMC_622_ cathode (thickness = ∼40 μm, Ø = 12 mm) was used as the positive electrode. Different stainless-steel spacers were used to adjust and maintain the cell stack pressure within the coin cell. Note that the pressure was increased by keeping the stack thickness constant while using thicker spacers. Detailed information about the calculation of the cell stack pressure can be found in the Supporting Information.

#### 2.4.2 Pouch Cells

For a comparison of the different applied external pressures and their impact on the cycling and rate performance, a two-electrode pouch-type cell setup was used. Li metal anode (thickness = ∼50 μm, 45 × 45 mm square-punched) was combined with NMC_622_ cathodes (thickness = ∼40 μm, 40 × 40 mm square-punched). A nickel tab was used as a current collector for the anode, whereas an aluminum tab was used as a current collector for the cathode. Both electrodes were separated by the selected SPE (thickness = ∼100 μm, 48 × 48 mm square-punched). Then, the cell stack was vacuum-sealed in pouch foil and sandwiched in between two metal plates, which were tightened with four screws and a torque wrench to apply reliable and reproducible external pressure on the cell stack. Detailed information about the pouch cell setup and how the pressure was applied and calculated are listed in the Supporting Information.

### 2.5 Electrochemical Measurements

#### 2.5.1 Measurement of the Ionic Conductivity

The ionic conductivity of the different SPEs was determined from EIS data using a Metrohm Autolab potentiostat. All of the samples were prepared by placing the polymer electrolyte film (Ø = 13 mm) between two stainless-steel blocking-type electrodes in a coin cell-type (CR2032) cell setup. In order to improve the interfacial contacts between electrodes and electrolyte, a preheated temperature loop was performed before cooling the samples to 0 °C. The measurements were carried out in a temperature range between 0 to 70 °C. An impedance measurement was conducted over a frequency range from 1 MHz to 100 mHz with an amplitude of 10 mV. A heating cycle comprised of a gradual temperature increase in 10 °C steps from 0 to 70 °C. After each temperature change, the cell temperature was held constant for two h prior to the acquisition of the impedance spectra. At a temperature of 70 °C, the heating profile was reversed and gradually cooled to 0 °C in 10 °C temperature steps. The corresponding ionic conductivities σ were derived according to eq 1.
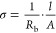
1*R*_b_ is the bulk electrolyte resistance that can be accessed from a Nyquist plot, *l* represents the electrolyte film thickness, and *A* is the film area.

#### 2.5.2 Potentiodynamic Experiments

All dynamic experiments were performed in a two-electrode cell setup with a VMP3 multichannel potentiostat of Biologic. All cells were conditioned at 60 °C for 12 h prior to the measurement.

For a determination of the LCD, a current ramp with a sweep rate of 1.0 μA s^–1^ was applied until a current density of 5.0 mA cm^–2^ or a cutoff voltage of 5.0 V was reached; a steep voltage increase indicates the actual LCD. Three cells were assembled and the LCD were reported as mean values, whereas only one representative cell is shown in the graph.

#### 2.5.3 Constant Current Cycling Experiments

All constant current cycling experiments were performed in a two-electrode cell setup by using an Arbin Battery Testing System. The cells were conditioned at 60 °C for 12 h before a current load was applied. Two cycles at 0.05 C, two cycles at 0.1 C, and two cycles at 0.05 C were conducted as the cell formation procedure prior to long-term cycling, and four cycles at 0.05 C were conducted as the formation procedure prior to the rate performance experiments. For long-term cycling experiments, the cells were cycled at a constant current of 0.2 C (60 μA cm^–2^) for 150 cycles. For the rate capability tests, the cells were cycled for four cycles with the same charge and discharge rate as indicated in the graph, and the stated capacity is normalized to the mean of the specific discharge capacity of the four cycles at 0.05 C.

Moreover, impedance spectra of the freshly assembled cells and the cells after formation and after cycling were collected using a Metrohm Autolab PGSTAT204 in a frequency range of 20 kHz to 1 Hz with an excitation amplitude of 10 mV. The impedance data were analyzed by invoking the software RelaxIS (rhd-instruments). Nyquist plots were fitted based on equivalent circuits, as indicated in the graph. Also, DRT analysis was performed in a frequency range of 20 kHz to 1 Hz, exploiting the regularization parameter of λ = 0.001.

### 2.6 Physicochemical Measurements

#### 2.6.1 Compression Test

The compressibility and mechanical properties of the different SPE were examined by using an Instron 5900 Series dual-column universal testing machine with 50 mm compression plates. The samples were prepared as mentioned before but with a thickness of 1.0 mm and a Ø°= 15 mm. All SPE membranes were placed on the lower compression plate and were equilibrated at 60 °C for 10 min. To allow for reliable and reproducible contact between the SPE and the compression plates, a preload of 1.0 N was applied before starting the measurements. The displacement rate of the compression test was controlled either by using a displacement (strain-controlled, 0.1 mm min^–1^ until a total force of 1.0 kN is reached) or force (stress-controlled, 1.0 N s^–1^ until a total force of 0.6 kN is reached). As a result, the compressive stress [MPa] was plotted against the compressive strain [%] and vice versa.

#### 2.6.2 Oscillatory Rheology

Rheological measurements were performed on a stress-controlled MCR 301 (Anton Paar) rheometer via oscillatory shear experiments. Sample membranes were prepared according to the previously mentioned technique but with a membrane thickness of 0.5 mm and diameter Ø°= 15 mm. For determining the overall storage (*G′*) or loss (*G″*) modulus, a frequency sweep from 0.1 to 100 rad s^–1^ at a constant amplitude of 0.1% was applied at 60 °C. For determining the yield stress (τ_*y*_) of the selected SPE an amplitude sweep from 0.01 to 100% with a fixed frequency of 0.1 rad s^–1^ was conducted at 60 °C. The yield stress is indicated in the respective graphs and is the beginning of the deviation from the linearity of the storage modulus. The flow point (τ_f_) is also indicated in the graph and is the intercept between the storage and loss modulus. A constant force of 1.0 N was applied to ensure good interfacial contact and reproducible pressure; the temperature was held constant for 5 min before applying the frequency or amplitude sweep. All the samples were measured under a nitrogen atmosphere.

## 3 Results and Discussion

### 3.1 Polymer Characteristics

**Figure 1:**
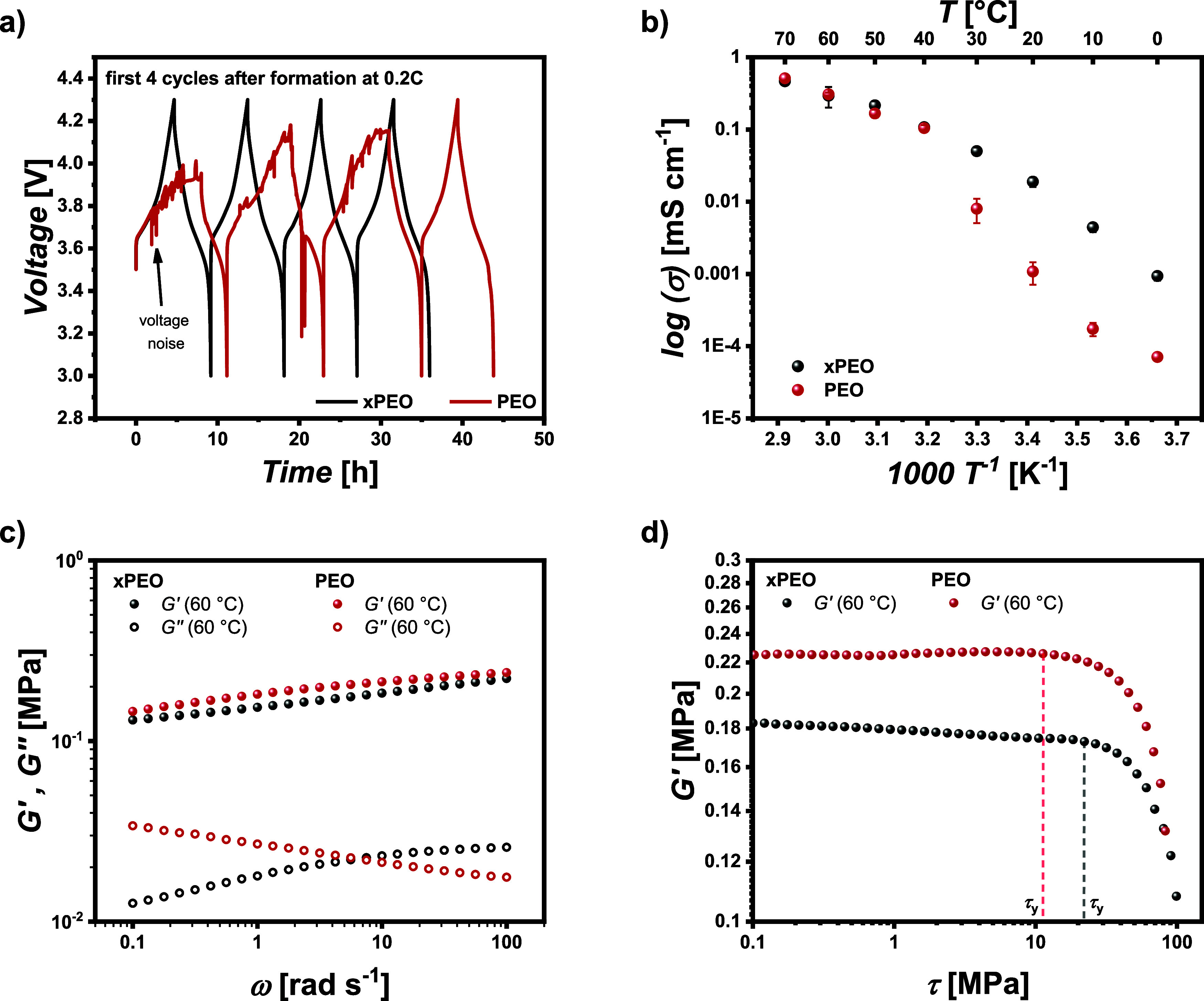
(a) Voltage profiles of NMC_622_||Li cells operated with cross-linked PEO (xPEO) and PEO after formation at 0.2C and 60 °C, (b) ionic conductivity of xPEO and PEO membranes at different temperatures, (c) storage (*G*′) and loss moduli (*G″*) as a function of the angular frequency (ω), and (d) amplitude sweep test to determine the yield stress (τ_*y*_) of xPEO and PEO.

PEO represents one of the most common polymers for polymer electrolytes due to its ability to dissolve Li salts and provide sufficient electrode wettability, yet its utilization in batteries is impeded by the occurrence of so-called “voltage noise” and compatibility challenges with high-voltage cathodes, primarily attributed to its debated limited oxidative stability. The voltage noise often results from Li dendritic growth and the occurrence of micro short circuits.^32,33^ The vulnerability of PEO-based polymers to microshorts depends on several parameters, e.g., molecular weight and thickness of the polymer membrane. Also, adjusting the cell setup has been reported to realize battery cell systems based on PEO electrolytes paired with high-voltage cathodes, suggesting to look at this strategic parameter more carefully.^34−36^ One mitigating strategy relies on the introduction of more rigid materials such as ceramics to physically delay or block Li dendrite penetration.^37,38^ Another approach involves the formation of a polymer network by cross-linking the polymer chains.^36,39^Figure 1a displays the voltage profiles of cells utilizing either PEO or cross-linked PEO (xPEO) as the SPE.

For the nonmodified PEO membrane, the occurrence of voltage noise prevents the cells from reaching their cutoff voltage of 4.3 V (instead limited by step-time), while the xPEO membrane can be cycled normally in the voltage range between 3.0 and 4.3 V. This eventually allows for the application of xPEO membranes in combination with NMC_622_ electrodes for investigations regarding external pressure. The overall ionic conductivity (Figure 1b) is not affected by cross-linking above an operating temperature of 40 °C, the polymer chains still offer sufficient movability to facilitate proper Li^+^ transport through the electrolyte. Both membranes have a similar ionic conductivity of 0.3 mS cm^–1^ at 60 °C, which is comparable to previous data reported for PEO-based electrolytes.^40^ At temperatures below 40 °C, cross-linking prevents the abrupt decrease in ionic conductivity observed for the PEO membrane by suppressing the formation of crystalline phases, thus providing a higher degree of amorphous phases for Li^+^ transport. The mechanical strength is a crucial factor in determining the possibility of a solid electrolyte to prevent Li dendritic growth or protrusion. A typical method includes the measurement of rheology by a frequency sweep with a fixed amplitude where no plastic deformation occurs (Figure 1c). Here, the storage moduli of both membranes are very comparable and in the range of 0.1 MPa at 60 °C with the loss moduli being much lower, thus, behaving like viscoelastic solids. Even though the storage modulus is around 4 orders of magnitude lower than necessary to in theory mechanically suppress Li dendritic growth, a positive effect on the morphology of Li metal deposits and the growth of Li globules through the electrolyte was demonstrated earlier.^41^ However, the comparable storage moduli of both membranes do not explain the observed differences in suppressed voltage noise during the cycling of the cells. As suggested by Chakraborty et al., the frequency sweep might not be the best method to determine the resistance against the growth of Li protrusions. Instead, they suggest considering the shear stress (τ) and the effect of yield stress (τ_*y*_), which is the value of the shear stress at the limit of linear viscoelastic regions, e.g., by performing an amplitude sweep.^42^ By this approach, a different behavior between PEO and xPEO can be observed (Figure 1d). The xPEO has a later limit of the linear viscoelastic region even though *G′* is lower compared to PEO, indicating a higher resistance against the rupture of bonds within the network. Since xPEO reduces the occurrence of dendritic Li-based micro short-circuits while increasing the limit of the linear viscoelastic region, it was applied for further evaluation of external pressure on the cycling performance of NMC622||Li cells.

### 3.2 Pouch Cell Setup for Applying External Pressure

**Figure 2:**
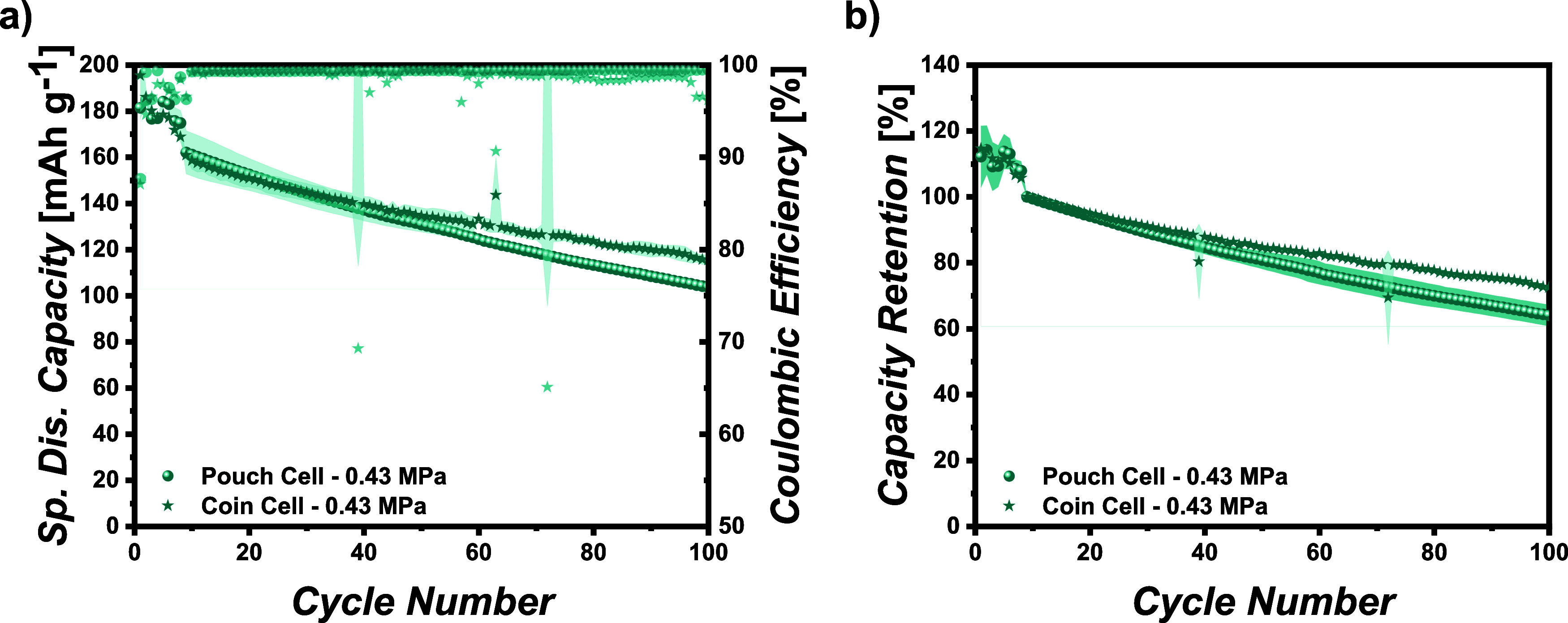
Comparison of long-term pouch and coin cell performance. (a) Specific discharge capacity and Coulombic efficiency vs cycle number and (b) capacity retention normalized to the first cycle at 0.2C after the formation vs cycle number.

We further decided to use a pouch cell setup for the electrochemical testing since the externally applied pressure can be much higher compared to a coin cell-type setup, where only a limited stack pressure range can be covered. The pouch-type as well as the coin cell-type setup, as well as the calculation of the applied external pressures, is described in the Supporting Information. To begin with, Figure 2a displays a long-term cycling experiment for coin cells and pouch cells at a stack pressure of 0.43 MPa to examine if the general cell performance of different setups is comparable.

Minor discrepancies are due to the different cell setups and the active electrode area. The coin cell setup displays a “noisier” capacity behavior, whereas the pouch cell setup with a 12-fold larger active electrode area can somehow compensate for electric current or temperature fluctuations during cycling.

In contrast, the capacity fade of the pouch cell setup is insignificantly increased compared to that of the coin cell setup. This might be explained by an inhomogeneous pressure distribution of the metal plates resulting in localized increased pressures and accelerated capacity fading, as discussed in the next section in detail. However, the general trend of both kinds of cells is comparable, rendering the previously mentioned discrepancies insignificant. Thus, the effect of various externally applied pressures can be analyzed in the pouch cell setup.

### 3.3 Long-Term Cycling Performance

**Figure 3:**
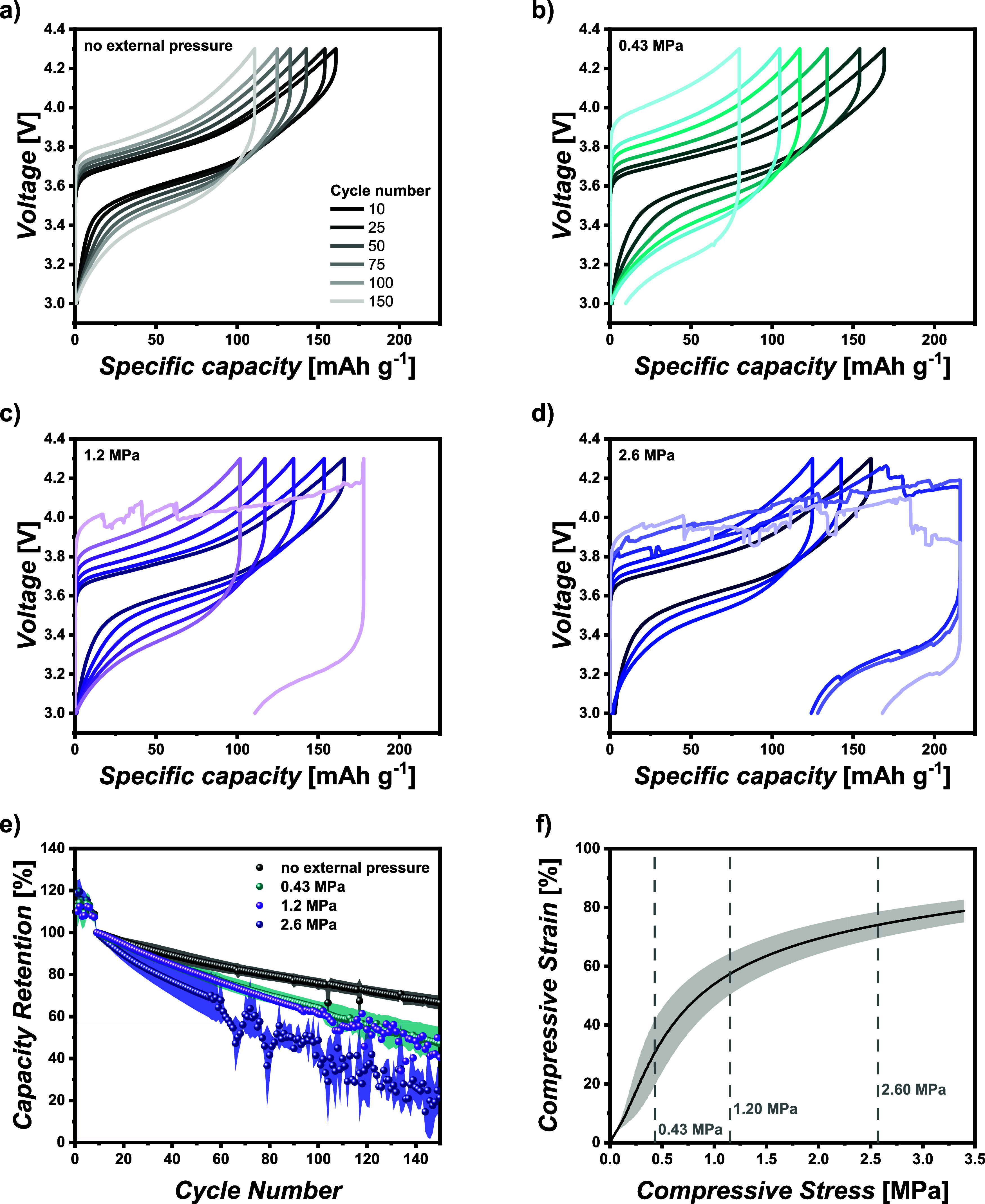
Long-term cycling performance of NMC_622_|xPEO|Li pouch cells with different external applied pressures at 0.2 and 60 °C. Voltage profiles of selected cycles with (a) “no external pressure”, (b) 0.43 MPa, (c) 1.20 MPa, (d) 2.60 MPa, (e) capacity retention of these pouch cells (referring to the 1st cycle after formation), and (f) compressive strain of xPEO membrane as a function of the compressive stress.

To examine the influence of external pressure on the long-term cycling performance of pouch cells, four different pressure levels were utilized: “no external pressure”, 0.43, 1.20, and 2.60 MPa. In the case of “no external pressure”, the pouch cells were cycled without metal plates and were only vacuum-sealed. Applying 0.43 MPa mimicked the pressure range typically found in coin cell setups for the given cell configuration, while 1.20 and 2.60 MPa represented higher external pressures. The voltage profiles of selected cycles are presented in Figure 3a–d, while Figure 3e illustrates the capacity retention during extended cycling. It is evident that increasing the external pressure yields decreased capacity retention. After 150 cycles, the cells without external pressure maintain 67% capacity retention, while the cells subjected to 2.60 MPa pressure retain only 22%. Moreover, poor capacity retention is accompanied by notable fluctuations in specific discharge capacity, starting from the 58th cycle. The voltage profile exhibits a “noisy” charging curve due to the formation of Li dendrites during these cycles. A similar behavior can be observed for the cells operated at a pressure of 1.20 MPa (Figure 3c, 150th cycle), while no voltage noise is observed in the other two setups. The cells subjected to 0.43 MPa experience a much higher increase in overvoltage and larger voltage hysteresis, likely due to the development of higher internal cell resistance compared to the cells without external pressure. The higher pressure is expected to enhance contacts with the active material, thereby resulting in accelerated electrochemical degradation and the continuous growth of interphases, leading to a higher IR drop and an accelerated voltage hysteresis. An extension of the cycle life by increasing the PEO membrane thickness was already demonstrated by Homann et al.^32^ Therefore, the cell stack thickness of these cells was measured after cycling. While the thickness over the whole electrode area varies, with slightly lower thickness at the edges and higher thickness in the middle, the mean thickness is in all cases lower than the one of fresh cells and decreases in the order: “no external pressure” > 0.43 MPa > 1.20 MPa > 2.60 MPa. The decrease in overall cell stack thickness after cycling is attributed to a deformation of the polymer membrane, which is the softest material in the stack and is pressed to the sides and inside the cathode during cycling. In order to investigate the impact of pressure on the polymer membrane, the compressive strain of xPEO was measured as a function of the compressive stress (Figure 3f)^36,39,43^ and the data were compared to the values applied in the pouch cells. Deformation of the polymer membrane occurs already below the lowest externally applied pressure of 0.43 MPa, but the compressive strain is only around 30%. For 1.2 and 2.60 MPa external pressure, the strain is 57% and 73%, respectively. Therefore, it can be assumed that a higher external pressure increases the deformation of the polymer membranes, thereby reducing the pathways for dendrites or Li protrusion to the cathode.

**Figure 4:**
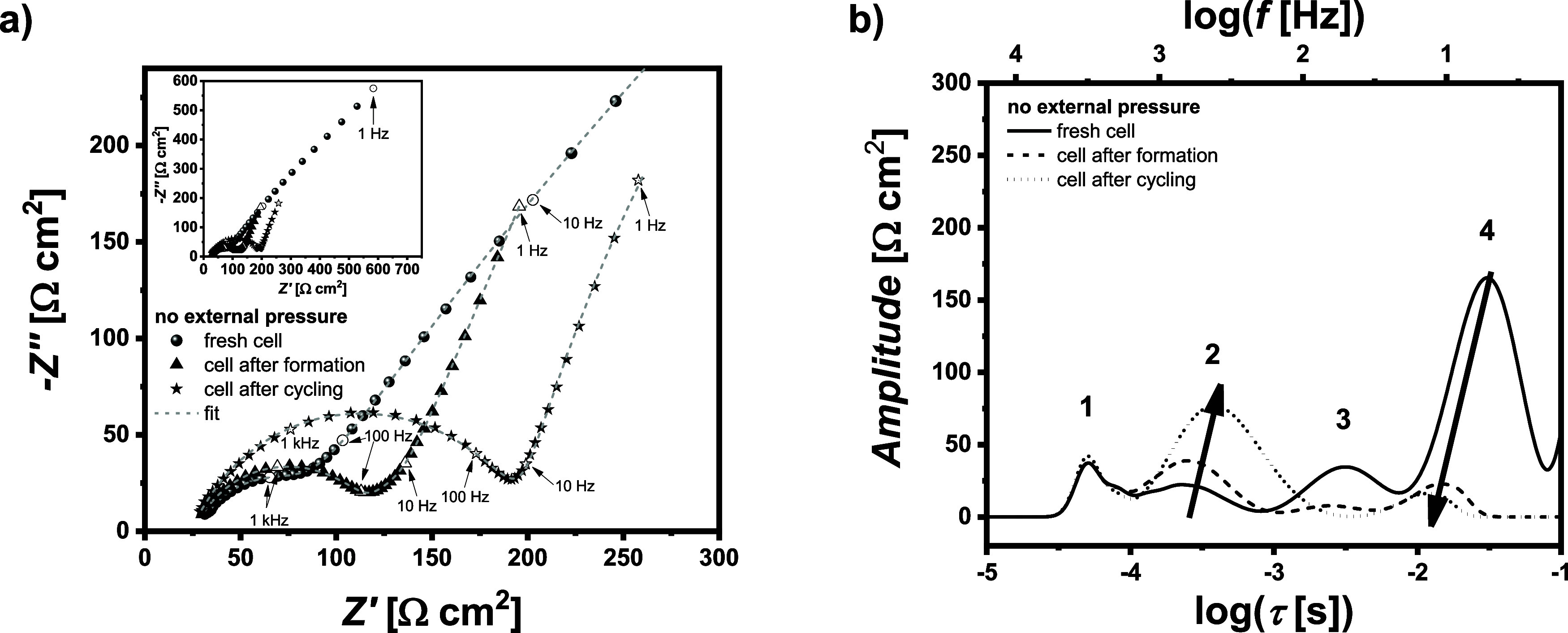
(a) Nyquist plots of the NMC622||Li cell without external pressure after cell assembly, after formation, and after cycling and (b) corresponding DRT analysis.

The cells without the application of external pressure have also been subjected to impedance analysis. The Nyquist plots after cell assembly, after formation, and after cycling are shown in (Figure 4a). In all cases, the presence of overlapping frequency domains results in depressed semicircles that cannot be completely separated. Notably, the fresh cells exhibit a smaller semicircle and a different low-frequency slope compared to the plots after formation and cycling. To gain a better understanding of the changes in the interface/interphase during cycling, the DRT analysis was conducted (Figure 4b). The rates of individual processes are related to distinct time constants τ (τ ∝ 1/*f*, *f* is the frequency), which can help to distinguish processes such as ion transport through the solid electrolyte interphase (SEI) and charge transfer processes.^44^

At lower frequencies of 0.1–1 Hz, a large peak can be detected for NMC_622_||Li cells which most likely reflects the solid-state diffusion inside the cathode as this peak disappears in Li||Li cells (Figure S3a,b). Here, we focused on the analysis of peaks in the frequency range of 1 Hz–20 kHz in which four different peaks with distinct time constants can be identified and are displayed in Figure 4b. The first peak at around τ = 5 × 10^–5^ s remains unchanged upon cycling and is likely related to the bulk properties (ionic conductivity) of the electrolyte or is the first contribution from the SEI layer. The second peak can be assigned to the SEI/CEI layers and increases over time, which indicates an unstable and growing SEI/CEI. While xPEO can be cycled in NMC_622_||Li cells, we still see a continuous capacity decrease over 150 cycles which is probably reflecting growing interfacial resistances and decomposition products.^45^ Finally, the third and fourth peaks (τ = 10^–3^–10^–1^ s) are in the typical range for charge transfer and double layer effects^44^ and are both decreasing after formation and cycling. Especially for the fourth peak, the fresh cells display a very large peak, which is substantially reduced after the formation and slightly shifted to lower time constants. The absence of external pressure may restrict the contact between the electrolyte and the electrodes, but during formation, charge transfer processes are improved. Additionally, lithium deposition during cycling increases the lithium surface area, further contributing to the observed phenomena.

### 3.4 Rate Performance

**Figure 5:**
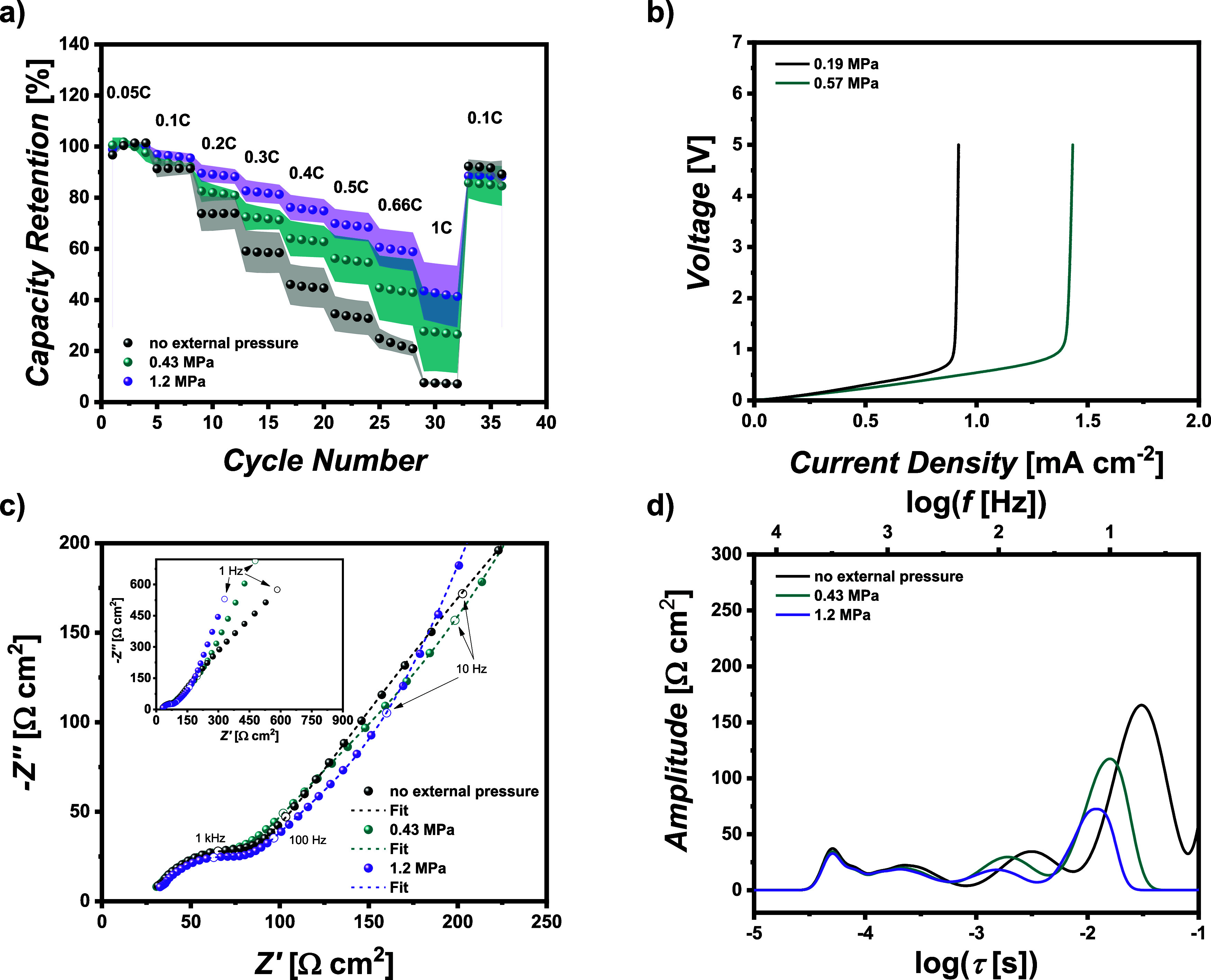
Investigation of the ion and charge transport behavior of the PEO-based pouch cells with varying external pressure. (a) Impact of the applied pressure on the rate capability of NMC_622_||Li full cells and (b) measurement of the limiting current density (LCD) with high and low pressure. (c) Nyquist plot of freshly assembled pouch cells and (d) corresponding DRT analysis.

Figure 5 shows different ion and charge transport investigations of xPEO SPE, while Figure 5a reveals the rate capability of cells with no applied external pressure in comparison to cells with an applied external pressure of 0.43 and 1.2 MPa. Note that the externally applied pressure was only varied up to 1.2 MPa for the rate capability investigations since cells with an elevated pressure of 2.6 MPa display severe voltage issues after just a few cycles of ongoing cycling.

It is obvious that an increase in external pressure leads to improved rate capability, resulting in a capacity retention of over 40% at 1C and 1.2 MPa, in contrast to less than 10% without external pressure. While the surface area remains consistent for all pouch cells, the effective contact area between the polymer electrolyte and electrodes is influenced by factors such as the roughness of the Li metal electrode and the penetration of polymer into the porous cathode, enhancing contact points with the active material. Hence, a well-designed interface becomes even more crucial.^46^ Increasing the pressure addresses interfacial issues and minimizes contact losses during cycling by augmenting and sustaining the overall contacts between the electrolyte and the electrode. This improvement enhances the ion and charge transfer effects, ultimately leading to an increased rate capability. To further corroborate this, the LCD was measured utilizing a fast-current scan in Li||Li symmetric cells (Figure 5b). A sharp increase in the voltage indicates the ion transport limit of the electrolyte. By increasing the external pressure from 0.19 to 0.57 MPa, the LCD is shifted from 0.88 ± 0.06 to 1.36 ± 0.11 mA cm^–2^. While, in theory, external pressure should not impact the LCD assuming an ideal contact between polymer and electrodes, the pressure does support sustaining the contacts between the polymer and the reshaping electrode surfaces during plating and stripping of Li. Also, EIS data of fresh cells were collected under various external pressures. Herein, we concentrate on the data of freshly assembled pouch cells to avoid misinterpretation of the impedance data due to side effects based on decomposition products or interphase contributions. As mentioned before, charge transfer processes are affected not only by the pressure but also by the formation of the cells. Figure 5c, d features the Nyquist plot and DRT analysis, respectively. By fitting the Nyquist plot (one example for the fit and the corresponding equivalent circuit is displayed in Figure S3d) the different components of the cell resistance, such as bulk electrolyte resistance (*R*_bulk_), resistance of interphases (*R*_SEI/CEI_) or charge transfer resistances (*R*_CT_) can be evaluated, as summarized in Table 2. By increasing the external pressure, the resistance of the electrolyte and the resistances of the interphases remain almost constant, whereas the charge transfer resistance is substantially reduced at higher external cell pressure. This agrees with the former results since the rate capability strongly depends on transport processes, which can macroscopically be characterized by the charge transfer resistance.

**Table 2:** Results of the Fitting of the Nyquist Plot Based on a Selected Equivalent Circuit^a^

	*R*_**bulk**_**(Ω cm**^**2**^**)**	*R*_**SEI/CEI**_**(Ω cm**^**2**^**)**	*R*_**CT**_**(Ω cm**^**2**^**)**
no external pressure	29	44	115
0.43 MPa	28	52	86
1.2 MPa	31	53	63

a *R*_bulk_ is the resistance of the solid polymer electrolyte (SPE); *R*_SEI/CEI_ is the resistance of the interphases; and *R*_CT_ is the charge transfer resistance.

Finally, a DRT analysis was performed based on the EIS data. The DRT analysis verifies elements of the fitting of the Nyquist plot. Referring to the literature, the first two peaks (τ < 10^–3^ s) of the DRT analysis can be attributed to interphases and the two remaining peaks (τ = 10^–3^ s–10^–3^ s) can mainly be attributed to charge transfer resistances.^44^ Note that the peak area mirrors the absolute value of the resistance and matches the results displayed in Table 2. The peak area in the region where the resistance of the interphases is dominant is nearly unaffected, indicating that SEI and CEI layers of fresh cells are not really affected by pressure, whereas notable changes in the peak area can be found in the region where charge transfer resistance predominates. Another important characteristic one can conclude from the DRT analysis includes the peak position regarding the relaxation time τ (*x*-axis). On the one hand, the peak position is the same for various pressures for peaks in the region where interphase resistances predominate. On the other hand, when the external pressure is increased, there is a meaningful shift to reduced relaxation times for peaks in the region, where the charge transfer resistance predominates. A reduced relaxation time implies an accelerated process.^47^

### 3.5 Impact of Mechanical Properties of the Selected SPE

In addition to PEO, we also investigated the influence of the external pressure on a second polymer. In this study, we utilized cross-linked GCD-PCL (xGCD-PCL), which had been previously employed in similar research.^31^ The caprolactone functional group present in the polymer contributes to increased Li^+^ transference numbers and sufficient Li^+^ conductivity.^31^

**Figure 6:**
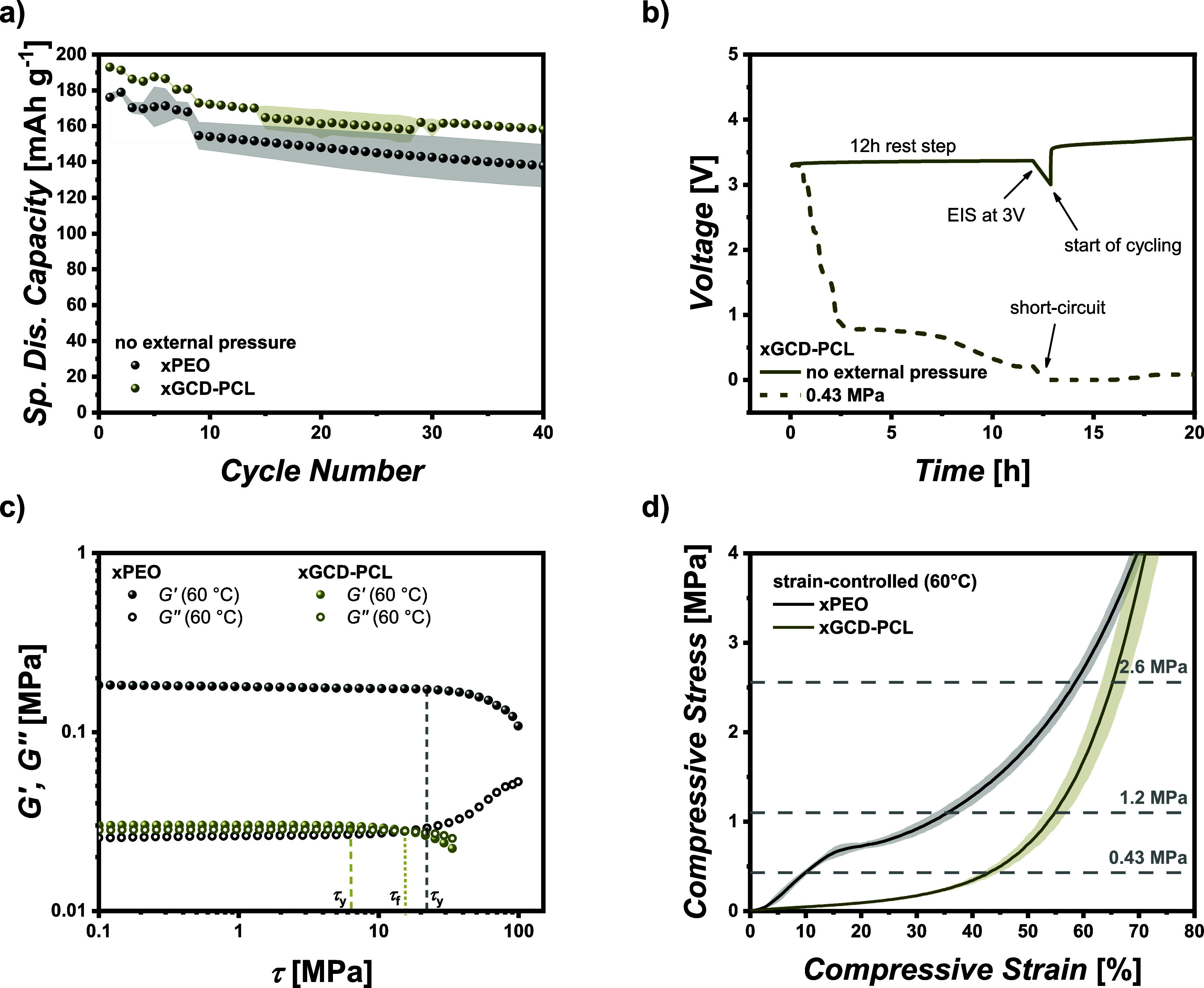
Influence of external pressure on the performance of cross-linked GCD-PCL (xGCD-PCL) in comparison to xPEO. (a) Specific discharge capacities of xGCD-PCL and xPEO membranes without external pressure, (b) voltage profiles of xGCD-PCL membranes without external pressure and after applying a pressure of 0.43 MPa, and (c) amplitude sweep test to determine the yield stress (τ_y_) of xGCD-PCL and (d) compressive stress of xGCD-PCL and xPEO membranes as a function of the compressive strain.

When examining NMC_622_||Li cells without the application of external pressure, the xGCD-PCL polymer membrane even slightly outperforms the xPEO membrane with 173 vs 155 mAh g^–1^ as specific discharge capacity at the first cycle after formation (Figure 6a). The capacity fade during cycling appears to be similar between the two membranes. However, when an external pressure of 0.43 MPa was applied, a short circuit occurred during the rest of the step before the formation process. The voltage profile of the rest step is depicted in Figure 6b, showing a continuous decrease until the cell voltage reaches almost 0 V. At the beginning of cycling, the cell is immediately shorted. Since there is no current flow during the rest step, the formation of lithium dendrites can be ruled out as a cause for the short circuit, suggesting that it is the result of membrane deformation and failure. Comparing the mechanical strength via measurement of the yield stress by means of rheology corroborates the observation that xGCD-PCL has limited mechanical strength (Figure 6c). *G′* and *G″* have almost equal values and the yield stress (τ_*y*_) is reached at a substantially reduced shear stress compared to xPEO. Besides, the existence of a flow point (τ_f_), which displays the shear stress at which *G′* intersects *G*″**, reflects that the polymer behaves more like a viscous liquid. Also, by monitoring the compressive stress as a function of the compressive strain (Figure 6d), the xGCD-PCL membrane is compressed quite easily without a strong increase in the compressive stress. In contrast, the xPEO membrane has a steeper increase of compressive stress when compressing the polymer. Overall, the xPEO membrane demonstrates higher resistance against compression and, hence, superior cycling performance when applying external pressure to the cells. While polymers such as xGCD-PCL might work well without or under only slightly applied pressure, they face severe problems at pressures that might also persist inside a coin cell. Therefore, the applied cell pressures should be optimized with respect to the polymer properties to achieve the best overall electrochemical performance.

## 4 Conclusions

**Figure 7:**
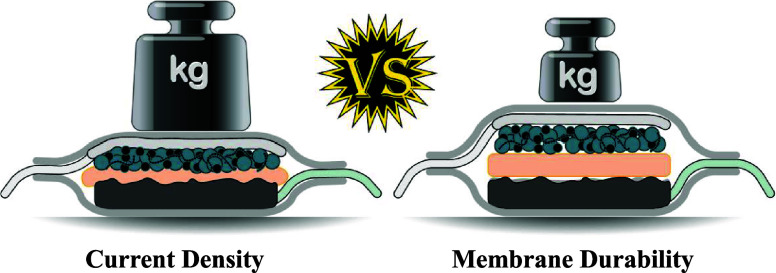
Graphical conclusion about the trade-off between rate capability and cell longevity.

In this study, we critically evaluated the impact of externally applied pressure (no external pressure, 0.43, 1.2, and 2.6 MPa) on the long-term performance and rate capability of NMC622|SPE|Li pouch cells (SPE: xPEO and xGCD-PCL). The application of high external pressure was demonstrated to accelerate the occurrence of voltage noise and cell failure, likely attributed to the limited mechanical properties of solid polymer electrolytes, resulting in the thinning of the polymer membrane during continuous cell operation. The softer xGCD-PCL membrane can be cycled only without external pressure in order to avoid plastic deformation, whereas the more rigid xPEO can withstand external pressures of up to 0.43 MPa. The rate capability is improved by applying external pressure due to an improved interface formation between the electrolyte and electrode, yielding higher LCD and accelerated transport processes. In conclusion, a higher external pressure enhances rate capability but intensifies capacity fading, while lower external pressure extends cell longevity at the expense of fast charge abilities (Figure 7). It is noteworthy that a more optimized external pressure may be determined through narrower external pressure steps, depending on which compromises between longevity and rate capability are defined as the optimum condition. Improving the mechanical stability of soft polymer electrolytes, e.g., by hybrid electrolytes employing mechanically more stable inorganic materials, block copolymers with a rigid block, or polymer blends containing a hard polymer, could allow an increased external pressure for higher current densities without limiting the cycle life. Sandwich-structured solid electrolytes involving a more rigid ceramic electrolyte in between a soft/melted polymer electrolyte for improved interphases, e.g., xGCD-PTMC, could also be a suitable solution, thus combining the advantages of both material classes. However, a comprehensive analysis of the properties of such modified solid electrolytes would be necessary to differentiate and understand the impact of external pressure which is beyond the scope of this study. Overall, we strongly recommend evaluating the mechanical properties of new polymer membranes concerning external pressure to determine the sweet spot of applicable current densities (fast charge) and the long-term durability of the considered cell designs. Nevertheless, the externally applied pressures in this work are considerably lower compared to the typically required pressures for the operation of ceramic (e.g., thiophosphate or argyrodite) electrolytes (≥5 MPa), highlighting the more practical implementation of polymers in commercial battery systems.

## Supporting Information Available

Pouch cell setup, calculation of the cell stack pressure in pouch cells, calculation of the cell stack pressure in coin cells, and electrochemical impedance spectroscopy (PDF)

## Abbreviations

PEO: poly(ethylene oxide)
GCD-PCL: α-cyclodextrin grafted poly(caprolactone)
BP: benzophenone
LiTFSI: bis(trifluoromethane)sulfonimide lithium salt
SPE: solid polymer electrolyte
EIS: electrochemical impedance spectroscopy
DRT: distribution of relaxation time
LCD: limiting current density
SEI: solid electrolyte interphase
