# Supplementary material for: External Pressure in Polymer-Based Lithium Metal Batteries: An Often-Neglected Criterion When Evaluating Cycling Performance?
Source: ACS Appl Mater Interfaces. 2024 Apr 22;16(17):21932–42. doi: 10.1021/acsami.4c02095 (PMC11071043; doi:10.1021/acsami.4c02095)
Supplement: Supplementary file 1 — am4c02095_si_001.pdf [file am4c02095_si_001.pdf]

Supporting Information

External Pressure in Polymer-Based Lithium Metal Batteries: An Often-Neglected Criterion When Evaluating Cycling Performance?

Philipp Röring[a,‡], *Gerrit Michael Overhoff*[a,‡], *Kun Ling Liu*[a], *Martin Winter*[a,b], *Gunther Brunklaus*[a,*]

a) Helmholtz-Institute Münster, IEK-12, Forschungszentrum Jülich GmbH, Corrensstraße 46, 48149, Münster, Germany.

b) MEET Battery Research Center / Institute of Physical Chemistry, University of Münster, Corrensstraße 46, 48149 Münster, Germany.

‡These authors contributed equally.

Email: g.brunklaus@fz-juelich.de

Table of Contents

Pouch Cell Setup 2

Calculation of the cell stack pressure in Pouch Cells 2

Calculation of the cell stack pressure in Coin Cells 5

Electrochemical Impedance Spectroscopy 7

Pouch Cell Setup

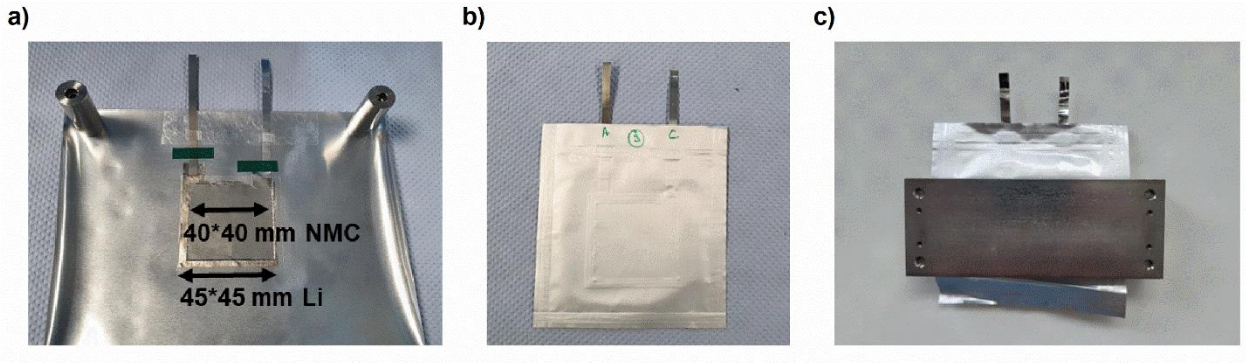

Figure S1: Pouch cell setup. a) cell stack before vacuum sealing, b) vacuum-sealed pouch cell and c) pouch cell sandwiched between two metal plates. The external pressure is applied by tightening the screws with a defined torque.

Derivation of the Cell Stack Pressure in Pouch cells

A Tekscan OEM Development Kit with a FlexiForce square-shaped (50 mm*50 mm) sensor was utilized to determine the applied external pressure in case of the single-layer pouch-type cells. The device was connected to a computer and controlled by FlexiForce Microview software. A three-point-calibration was performed prior to accessing the applied pressures. For calibration, three different known weights (**Table S1**) were put on the sensor and the digits (a.u.) were count.

Table S1: Mass of the different weights of the three-point-calibration.

Calibration Point	Mass [kg]
1	21.5
2	35.0
3	67.4

Thus, the to be determined pressure applied to the cells is related to a specific mass in kilograms. According to equation (1) the pressure p can be obtained from:

$$p = \frac{m \cdot g}{A} \tag{1}$$

with m being the mass of the weight, g being the gravity acceleration ($g = 9.81$ m s^{-2}) and A the overall area onto which the pressure is applied ($A = 1600$ mm^2). The Tekscan OEM development kit derived the mass as a mean value of 100 data points. To minimize the impact of various metal plates and exact position of the pouch cell sandwiched in between the metal plates, three different plates were used and the masses were obtained from the mean of three different test series for each tightening torque. A series of eight tightening torques were applied on the sensor and the pressure was calculated according to equation (1). The results are displayed in **Table S2**. Note that the standard deviation increases for higher tightening torques as the corresponding weights fall significantly outside the calibration region while for lower tightening torques, a higher accuracy can be assumed. The calculated pressure was then plotted against the tightening torque (**Figure S2**) and the curve was fitted by the exponential function

$$y = a - b \cdot c^x \tag{2}$$

with a = 3.25, b = 3.06 and c = 0.69. Expression (2) was invoked as fit function since it represents the data points very well with a coefficient of determination R^2 of 0.996.

Table S2: Parameter for calculating the external pressures, which are applied onto the pouch-type cells by tightening the screws with a specific torque.

**Tightening Torque [Nm]**	**Mass [kg]**	**Pressure [N mm^{-2} = MPa]**
0.10	40 ± 7	0.24 ± 0.04
0.20	61 ± 10	0.38 ± 0.06
0.30	91 ± 11	0.56 ± 0.07
0.46	125 ± 21	0.77 ± 0.1
1.0	188 ± 35	1.2 ± 0.2

2.0	288 ± 47	1.8 ± 0.3
3.0	364 ± 49	2.2 ± 0.3
4.0	424 ± 52	2.6 ± 0.3

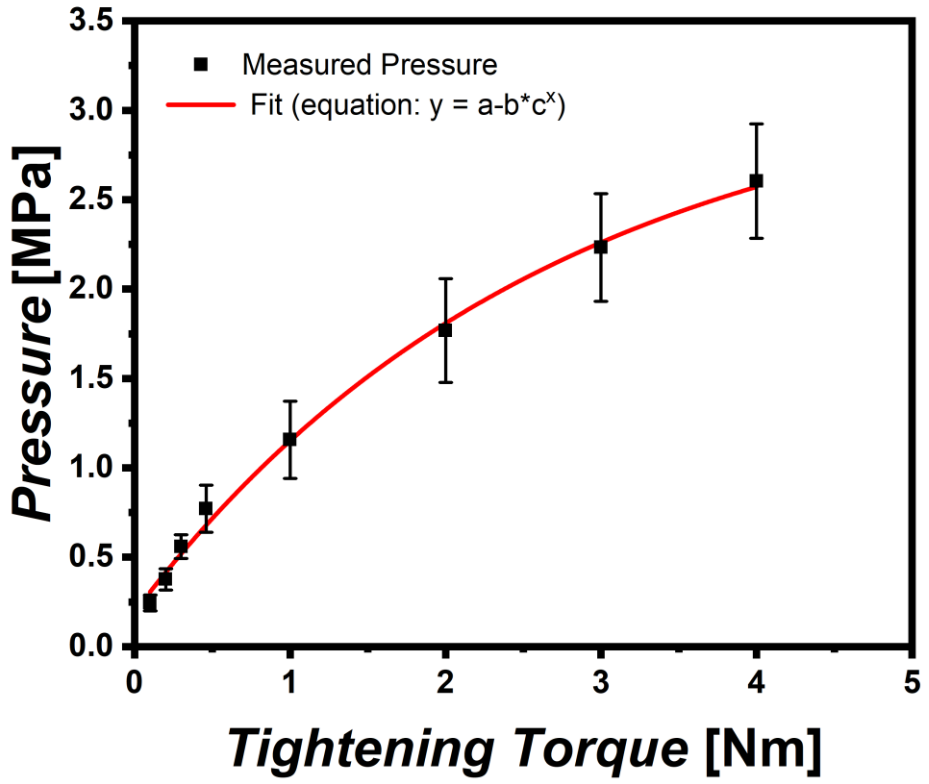

Figure S2: Pressure against the torque used to apply external pressure onto the pouch-type cells. The red curve shows the exponential fit to obtain the actual pressures within the considered range of torques.

With equation (2), the pressure of each torque can be calculated. For comparison of the impact of pressure on the achievable battery performance, three different externally applied pressures were set (**Table S3**). The selected values are adequately spaced apart to prevent overlapping regions, considering the increased standard deviations observed at higher tightening torques. Notably, a

pressure of 0.43 MPa, which results from a torque of 0.21 Nm, is similar to established pressures in coin cell setups, which is described in more detail in the following section. In addition, pouch-type cells were operated without metal plates, labelled 'No external pressure', where only the vacuum-sealing ensures contact between electrodes and electrolyte.

Table S3: Externally applied pressures resulting from different tightening torques.

Tightening Torque [Nm]	Pressure [MPa]
0.21	0.43 ± 0.06
1.0	1.2 ± 0.2
4.0	2.6 ± 0.3

Calculation of the Cell Stack Pressure in Coin Cells

According to expression (3) the pressure *p* can be calculated as:

$$p = \frac{F}{A} = \frac{D \cdot \Delta L}{A} \tag{3}$$

where *F* is the force and *A* is the area onto which the force is applied. The force can be determined from the spring constant *D* ($D = 100$ N mm^{-1}) and the deflection of the spring ΔL. *D* was measured invoking a Zw*i*ckRoell spring testing machine. Thus, a force of F = 50 N was applied and the corresponding deflection ΔL of the spring was measured. Here, a deflection of $\Delta L = 0.5$ mm resulted as an average from ten measurements. According to equation (4), the respective spring constant was obtained as $D = 100$ N mm^{-1}.

$$D = \frac{F}{\Delta L} \tag{4}$$

ΔL of the spring within the crimped cells were derived according to expression (5) by subtracting the thickness of the individual cell components (**Table S4**) from the thickness of the crimped cells, being h_{cell} = 3.2 mm for CR2032-type cells.

$$|\Delta L| = h_{\text{cell}} - (h_{\text{case}} + h_{\text{spacer}} + h_{\text{an.}} + h_{\text{SPE}} + h_{\text{cat.}} + h_{\text{spacer}} + h_{\text{spring}} + h_{\text{case}}) \tag{5}$$

Table S4: Thickness of the cell components required to calculate the deflection of the spring when the cell is crimped.

**Individual cell component**	***L* (thickness) [mm]**
lower case	0.25
lower spacer	1.00
anode	0.05
solid polymer electrolyte	0.10
cathode	0.04
upper spacer	0.75
spring	1.25
upper case	0.25

In practice, the cell stack pressure can be varied by using different lower or upper spacers. Herein and according to equation (3), when using a lower spacer with L = 1.0 mm, an upper spacer with L = 0.75 mm and an area of A = 113 mm^2, corresponding cell stack pressures of p = 0.43 MPa were established.

Electrochemical Impedance Spectroscopy (EIS)

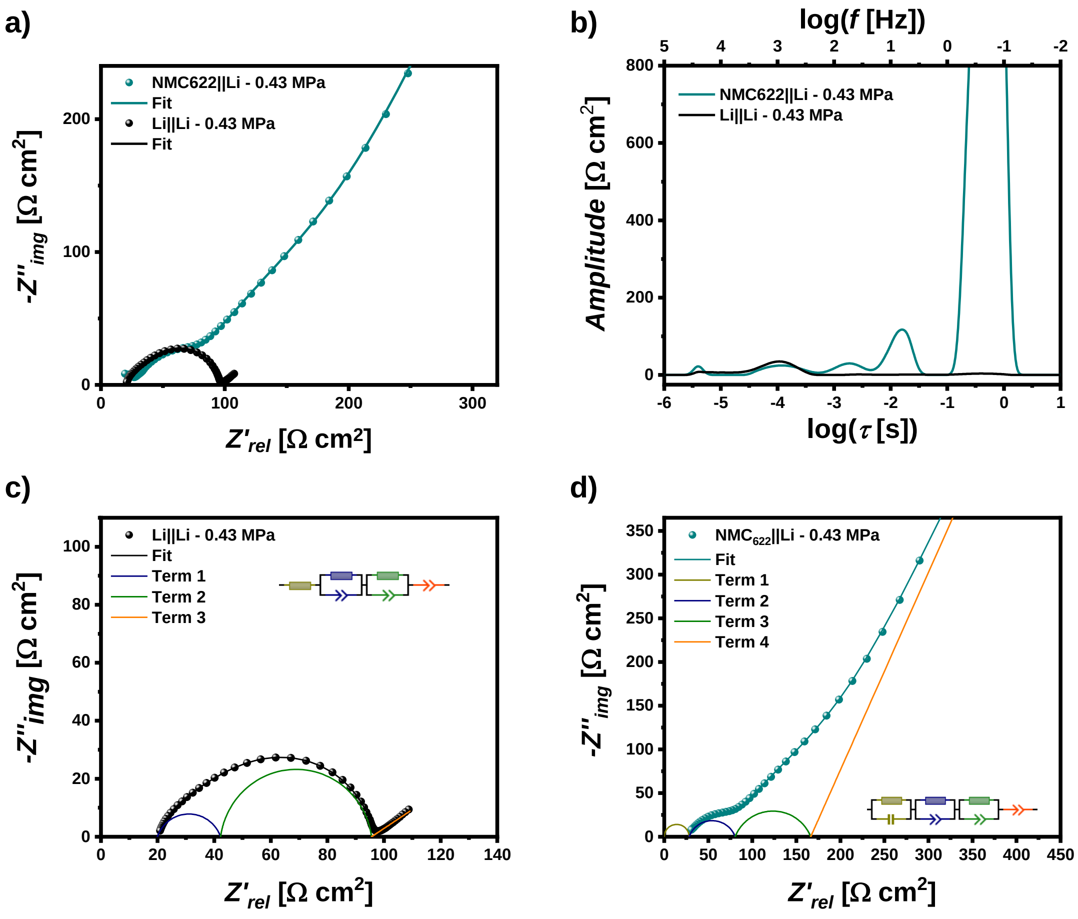

Figure S3: a) Nyquist plots of NMC_{622}||Li and Li||Li symmetric cells operated with xPEO, b) corresponding DRT analysis and fitted equivalent circuits in case of c) Li||Li- and d) NMC_{622}||Li cells.

Figure S3a) and **b)** exhibit the EIS data and the corresponding DRT analysis of Li||Li symmetric coin cells compared to NMC_{622}||Li pouch-type cells. The first intercept of the semi-circle with the x-axis is in both cell setups highly similar and displays bulk electrolyte resistances of the xPEO membranes. Interphase resistances and charge transfer resistances differ because of different cell setups and cell chemistries. Moreover, the DRT analysis (**Figure S3b)**) reflects that most of the

contributions to charge transfer resistances are in fact resulting from the porous cathodes. While peak position and peak area, in regions where electrolyte and interphase resistance predominate, are comparable in case of both cell setups, there are only minor peaks for Li||Li symmetric cells and superior peaks in case of NMC_{622}||Li cells in the respective region where charge transfer resistance predominates. Also, **Figure S3c)** and **d)** exhibit exemplary equivalent circuit fits of the impedance data of Li||Li coin cells and NMC_{622}||Li pouch-type cells. The different contributions of the fit as well as the equivalent circuits are marked with different colors.

Table S5: Fit parameters of the invoked elements of the equivalent circuit model displayed in **Figure S3d)**.

Equivalent circuit fit	NMC622||Li – 0.43 MPa, fresh
Resistance 1 / Ω cm^2	28.2
Capacitance 1	4.8E-06
Resistance 2 / Ω cm^2	52.3
Pseudo capacitance 2	0.0043
CPE factor α_2	0.78
Resistance 3 / Ω cm^2	85.8
Pseudo capacitance 3	0.068
CPE factor α_3	0.76
Pseudo capacitance 4	0.086
CPE factor α_4	0.74

In case of the considered NMC_{622}||Li cells, the determined resistances reflecting the semi-circles amount to 28.2 Ω cm^2 (yellow), 52.3 Ω cm^2 (blue) and 85.8 Ω cm^2 (green), respectively, accounting for the electrolyte resistance, SEI resistance and charge transfer resistance. Note that

comparable values were recently reported by Yusim et al. who studied the interfacial stability on the cathode side depending on the upper cut-off voltage of NMC|PEO|Li cells.[1]

References:

[1]: Yusim, Y.; Hunstock, D. F.; Mayer, A.; Bresser, D.; Passerini, S.; Janek, J.; Henss, A. Investigation of the Stability of the Poly(ethylene oxide)|$LiNi_{1-x-y}Co_xMn_yO_2$ Interface in Solid-State Batteries *Adv. Mater. Interfaces* **2023**, 2300532.
